# Impact of cardiorespiratory rehabilitation program on submaximal exercise capacity of Tunisian male patients with post-COVID19: A pilot study

**DOI:** 10.3389/fphys.2022.1029766

**Published:** 2022-09-28

**Authors:** Emna Toulgui, Wafa Benzarti, Chiraz Rahmani, Sana Aissa, Ines Ghannouchi, Asma Knaz, Amani Sayhi, Sana Sellami, Khaoula Mahmoudi, Sonia Jemni, Imene Gargouri, Abdelaziz Hayouni, Walid Ouanes, Achraf Ammar, Helmi Ben saad

**Affiliations:** ^1^ Department of Physical Medicine and Rehabilitation, Sahloul Hospital, Sousse, Tunisia; ^2^ Department of Pneumology, Farhat HACHED Hospital, Sousse, Tunisia; ^3^ Research Laboratory “Heart Failure, LR12SP09”, Faculty of Medicine of Sousse, Hospital Farhat HACHED of Sousse, University of Sousse, Sousse, Tunisia; ^4^ Department of Training and Movement Science, Institute of Sport Science, Johannes Gutenberg-University Mainz, Mainz, Germany; ^5^ Interdisciplinary Laboratory in Neurosciences, Physiology and Psychology: Physical Activity, Health and Learning (LINP2), UPL, Paris Nanterre University, UFR STAPS, Nanterre, France; ^6^ High Institute of Sport and Physical Education of Sfax, University of Sfax, Sfax, Tunisia

**Keywords:** handicap, health status, lung function test, SARS-Cov-2, walking, 6MWT

## Abstract

Post-COVID19 patients suffer from persistent respiratory, cardiovascular, neurological, and musculoskeletal health complaints such as dyspnea, chest pain/discomfort, and fatigue. In Tunisia, the potential benefits of a cardiorespiratory rehabilitation program (CRRP) after COVID19 remain unclear. The main aim of this study was to evaluate the impact of a CRRP on submaximal exercise capacity, evaluated through the 6-min walk test (6MWT) data in post-COVID19 Tunisian patients. This was a cross-sectional study including 14 moderate to severe COVID19 patients aged from 50 to 70 years. CRRP was performed after the end of patients’ hospitalization in COVID19 units for extensive or severe extents of COVID19. Dyspnea (modified medical research council), spirometry data, handgrip strength values, 6MWT data, and 6-min walk work (i.e., 6-min walk distance x weight) were evaluated 1-week pre-CRRP, and 1-week post-CRRP. CRRP included 12 sessions [3 sessions (70 min each)/week for 4 weeks]. Exercise-training included aerobic cycle endurance, strength training, and educational sessions. Comparing pre- and post- CRRP results showed significant improvements in the means±standard deviations of dyspnea by 1.79 ± 0.80 points (*p* < 0.001), forced expiratory volume in one second by 110 ± 180 ml (*p* = 0.04), 6-min walk distance by 35 ± 42 m (*p* = 0.01), 6-min walk work by 2,448 ± 3,925 mkg (*p* = 0.048), resting heart-rate by 7 ± 9 bpm (*p* = 0.02) and resting diastolic blood pressure by 6 ± 10 mmHg (*p* = 0.045). In Tunisia, CRRP seems to improve the submaximal exercise capacity of post-COVID19 patients, mainly the 6-min walk distance and work.

## 1 Introduction

The coronavirus disease 2019 (COVID19) pandemic has overburdened healthcare systems and it poses a threat to the global economy and social disruption (Bessis, 2020). COVID19 is a respiratory infection with multisystem manifestations, affecting the respiratory, cardiovascular, neurological, and muscular systems (Xie et al., 2022). Several symptoms (e.g., dyspnea, dysrhythmias, stroke, headache, myalgia, and asthenia), and complications (e.g., respiratory failure, acute myocardial injury, thromboembolic events) have been reported for acute-COVID19 (Long et al., 2020; Murk et al., 2021). In post-acute COVID19, 40%–90% of patients continue to manifest symptoms for months, and the disease is named “long-COVID19” (Lopez-Leon et al., 2021). The “long-COVID19,” also called “post-acute-COVID19” or “persistent-COVID19 symptoms,” has various clinical manifestations affecting several systems, mainly the respiratory, cardiovascular, neurological, and muscular systems (e.g., dyspnea, post-activity polypnea, cough, chest pain/discomfort, resting tachycardia, fatigue), and alters the nutritional status (e.g., weight-loss) (Lopez-Leon et al., 2021; Skjorten et al., 2021; Ali et al., 2022; Ghram et al., 2022). Several studies have reported persistent physical impairments following hospital discharge (e.g., reduced forced expiratory volume in one second (FEV_1_) and forced vital capacity (FVC) (Zhao Y. M. et al., 2020), decreased handgrip strength (HGS) (Cheval et al., 2021), and exertional dyspnea (modified medical research council (mMRC)) (Huang et al., 2021). In one study, the prevalence of musculoskeletal health complaints in “long-COVID19” patients was high at 38.7% (Ali et al., 2022). A reduced 6-min walk distance (6MWD) was reported by Huang et al. (2021), and it seems that 3 months after hospital discharge, one-third of long-COVID19 patients had a peak oxygen consumption <80% (Skjorten et al., 2021). Since survivors of moderate to severe COVID19 are significantly impaired in all activities of daily living (Skjorten et al., 2021), rehabilitation strategies are needed to improve post-COVID19 outcomes in this population. (Demeco et al., 2020; Hermann et al., 2020; Liu K. et al., 2020; Betschart et al., 2021; Bouteleux et al., 2021; Daynes et al., 2021; Gloeckl et al., 2021; Puchner et al., 2021; Spielmanns et al., 2021). The cardiorespiratory rehabilitation program (CRRP) is the cornerstone in the management of chronic cardiorespiratory diseases, and its benefits are well demonstrated (Ben Saad et al., 2008; Jenkins et al., 2018; Rezende Barbosa et al., 2018).

In COVID19, CRRP is a new management axis, and studies related to its impact on patients’ capacities are scarce (Demeco et al., 2020; Hermann et al., 2020; Liu K. et al., 2020; Betschart et al., 2021; Bouteleux et al., 2021; Daynes et al., 2021; Gloeckl et al., 2021; Puchner et al., 2021; Spielmanns et al., 2021). Two systematic reviews including almost 33 studies demonstrated the feasibility and efficiency of CRRP in the management of post-COVID19 patients (Demeco et al., 2020; Dixit et al., 2021). Almost all 33 retained studies were conducted in specialized rehabilitation units from industrialized countries. In low-income countries, such as Tunisia, the potential benefits of CRRP after COVID19 is unclear. Indeed, in these countries, CRRP centers are rare, no specialized rehabilitation equipment is available, and too few COVID19 patients have access to CRRP.

The objective of the present study, conducted in Tunisia, was to evaluate the impact of an ambulatory CRRP on perceived dyspnea (mMRC), spirometric, HGS, and 6-min walk test (6MWT) data. CRRP will be considered “efficient” if the delta CRRP changes (ΔCRRP = post-CRRP value minus pre-CRRP value) in the 6MWD and dyspnea mMRC scale exceed the recommended minimal clinically important differences (MCIDs) for respiratory chronic diseases [i.e., MCID = 30 m for 6MWD (Singh et al., 2014), MCID = one point for dyspnea (mMRC) (Crisafulli and Clini, 2010)].

## 2 Patients and methods

**FIGURE 1:**
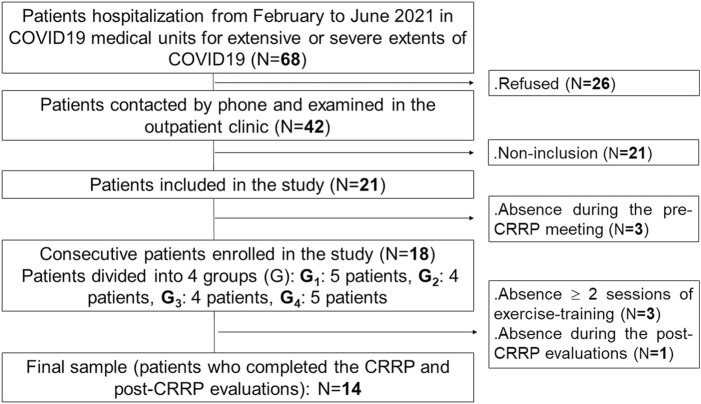
Study protocol. COVID19: coronavirus disease 2019. CRRP, cardio-respiratory rehabilitation program.

This study is part of a project involving two parts. The first part constitutes the aim of this study. The second part will be the evaluation of the impact of CRRP on social disadvantage (i.e., physical activity, psychological data, health-related quality of life). Figure 1 details the present project flowchart.

### 2.1 Study design

This was a cross-sectional study conducted by a multidisciplinary team, including the following three departments: the department of pulmonology, the department of physiology and functional explorations (Farhat HACHED hospital, Sousse, Tunisia), and the department of physical medicine and rehabilitation (Sahloul hospital, Sousse, Tunisia). This study was approved by the medical and research ethics committee of Farhat HACHED Hospital (Approval number FH2502/2021). Written informed consent was obtained from all patients after receiving an explanation of the study. This study was performed from February 2nd to September 26th 2021, including the Ramadan month (from April 13th to May 13th 2021). The period from February to June 2021 was reserved for the recruitment of COVID19 patients. The period from April to September 2021 was reserved for the practice of CRRP. The CRRP was performed at least 2 months after the end of the hospitalization in COVID19 units.

During the present study period (i.e., February 2nd to September 26th 2021), Tunisia decided to declare a 1-week nationwide lockdown starting from May 9th to May 16th 2021. During all the study steps, all recommended preventive measures to fight against COVID19 transmission (e.g., physical distancing of at least 1 m, wearing a fitted facemask properly, and cleaning hands frequently with alcohol-based hand rub or soap and water) were applied.

### 2.2 Study population

The source population was COVID19 patients living in Sousse (Tunisia) who needed hospitalization in a medical facility. The target population was patients hospitalized in COVID19 units of the aforementioned departments of pulmonology and physical medicine from February to June 2021 (Figure 1).

The following inclusion criteria were applied: confirmed diagnosis of COVID19, male patients, age >50 years, and chest computed tomography during the hospitalization period showing an extensive/severe extent of parenchymal lung injury (Revel et al., 2020). The applied exclusion criteria were: 1) COVID19 patients admitted in an intensive care unit; 2) contra-indications to 6MWT (Singh et al., 2014) [e.g., signs of unstable angina or myocardial infarction within the previous month, resting heart-rate ≥ 120 bpm, systolic blood pressure (SBP) ≥ 180 mmHg, diastolic blood pressure (DBP) ≥ 100 mmHg)]; 3) contra-indications to spirometry (Miller et al., 2005); and 4) orthopedic, rheumatologic, or muscular history, which may interfere with walking or HGS. Absence during two or more exercise-training sessions or the post-CRRP evaluation session was applied as an exclusion criterion.

### 2.3 Sample size

The sample size (N) was calculated according to the following predictive equation (Serhier et al., 2020): N = (Z_α_ p (1-p))/i^2^, where “**Z**_**α**_” is the normal deviates for type I error (equal to 1.28 for 90% confidence level), “***p***” is the percentage of improvement of the main outcome (i.e., 6MWD) post-CRRP in COVID19 patients; and « ***i*** » is the precision (*i* = 0.15). According to a Chinese study (Liu K. et al., 2020), the 6MWD of trained COVID19 patients (*n* = 36, mean age: 69.4 years) was improved by 30.5% (*p* = 0.305) [went from 163 ± 72 (pre-CRRP) to 212 ± 82 m (post-CRRP)]. The application of the above-mentioned data in the predictive equation gave a sample size of 15 COVID19 patients. Assumption of 10% of absence during the exercise-training sessions or the post-CRRP evaluation session gave a revised sample of 17 COVID19 patients [17 = 15/(1–0.10)].

### 2.4 Coronavirus disease 2019 diagnosis and extent evaluation

COVID19 diagnosis was confirmed by reverse transcriptase-polymerase chain reaction (RT-PCR) (Jamil et al., 2020). All patients underwent chest-computed tomography. The following two classifications were applied: 1) chest computed-tomography classification, including the following five levels based on the extent of parenchymal lung injury: *absent* or *minimal* (<10%), *moderate* (10%–25%), *extensive* (25%–50%), *severe* (50%–75%), and *critical* (>75%) (Revel et al., 2020), and 2) clinical classification (WHO, 2021), including the following four levels: *mild*, *moderate*, *severe*, and *critical*.

### 2.5 Applied protocol

**FIGURE 2:**
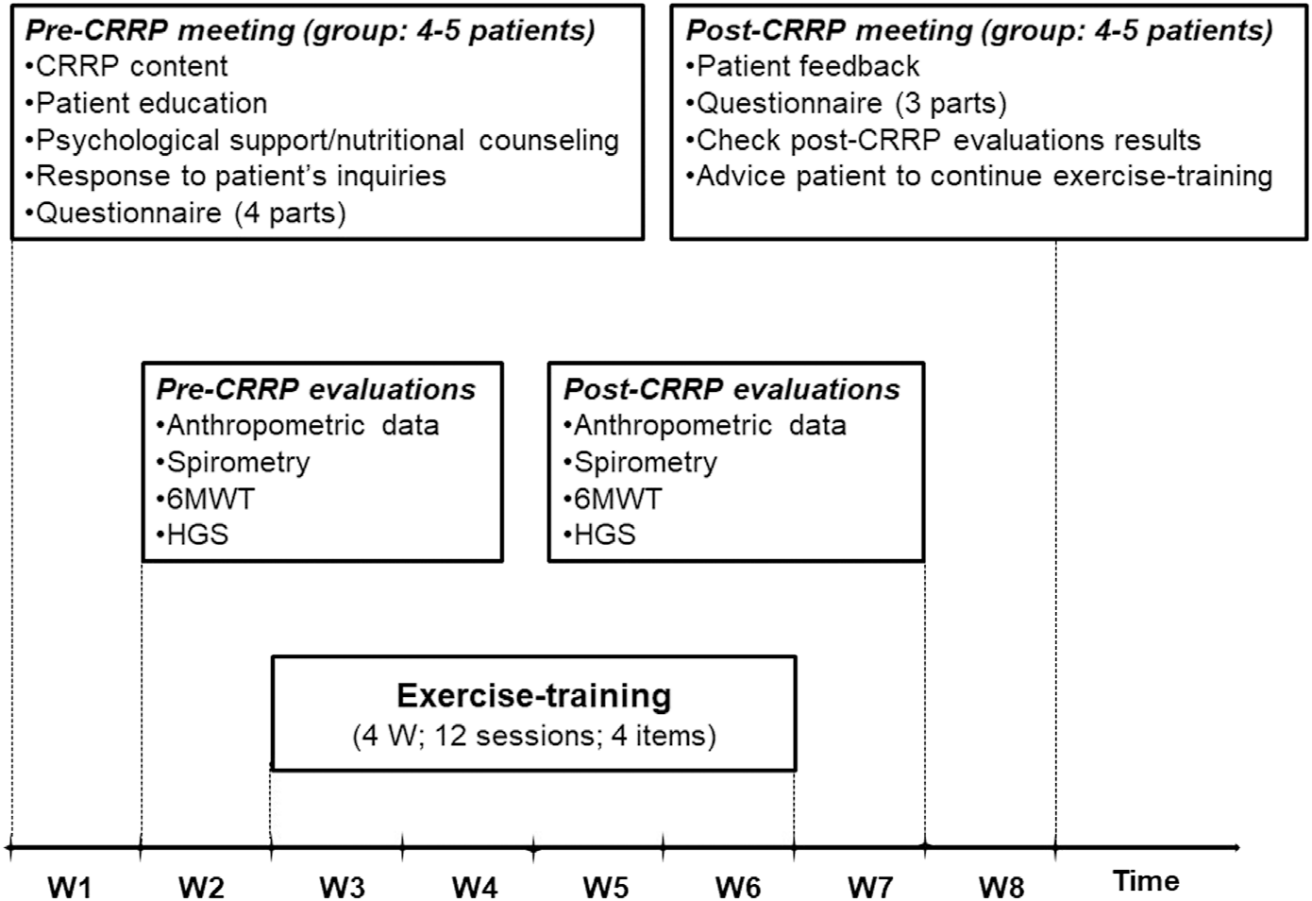
Cardiorespiratory rehabilitation program (CRRP). HGS, handgrip strength; W, week; 6MWT, 6-min walk test.

The components of the CRRP were “derived” from previous international recommendations for COVID19 CRRPs (Barker-Davies et al., 2020; Carda et al., 2020; Spruit et al., 2020; Zhao H. M. et al., 2020), and from the American societies of cardiology and sports medicine recommendations for the practice of physical activity in chronically ill patients aged over 50 years (Nelson et al., 2007). Once four to five consecutive patients agreed to participate in the CRRP, they formed one group (Figure 1), perform the recommended tests, and begin the CRRP. Figure 2 summarizes the five steps of the study.

#### 2.5.1 First step: Pre-CRRP meeting

The first step consists of a pre-CRRP meeting between two physicians (*ET* and *WB* in the authors’ list) and a group of four to five COVID19 patients. During this step, the following five actions were performed: 1) explanation of the CRRP content and its progress; 2) when applicable, education about how to manage comorbidities (e.g., diabetes-mellitus, arterial-hypertension), and encouraging smoking cessation; 3) psychological support (e.g., management of emotional distress, post-traumatic stress disorder, and strategies for coping with COVID19) (Simpson and Robinson, 2020), and nutritional counseling (Ghram et al., 2022); 4) response to patients’ inquiries; and 5) filling in the questionnaire.

The questionnaire was prepared in the local Arabic dialect by two trained physicians (*ET* and *WB* in the authors’ list). For each patient, the questionnaire was repeated by the same interviewer pre- and post- CRRP. The duration of the questionnaire was approximately 30 min for each patient. The questionnaire includes four parts. The first part (i.e., a general questionnaire), derived from the American thoracic society questionnaire (Ferris, 1978), was performed only pre-CRRP, and it involved clinical (e.g., lifestyle habits, medical history) and COVID19 (e.g., date of RT-PCR, hospitalization, number of days pre-CRRP, treatment, imaging) data. Cigarette smoking was evaluated in pack-years, and patients were classified into two groups [i.e., non-smoker (<5 pack-years), and smoker (≥5 pack-years)]. Hospital stay is the number of days of hospitalization for COVID19 management. The number of days pre-CRRP represents the number of days between COVID19 diagnosis (day of RT-PCR) and the first day of the onset of exercise-training. Dyspnea was assessed (pre- and post- CRRP) using the mMRC scale (Fletcher et al., 1959). The latter is a self-rating scale that measures the disability caused by breathlessness in daily activities (Mahler and Wells, 1988). This scale ranges from 0 to 4, where “0” is no breathlessness, except on strenuous exercise; and “4” is too breathless to leave the house, or breathless when dressing or undressing (Mahler and Wells, 1988). The remaining three parts of the questionnaire were reserved to explore the level of physical activity, current presence and tendency to anxiety or depression at the time of evaluation, and health-related quality of life. The data of the last three parts of the questionnaire will be explored in the second part of the project.

#### 2.5.2 Second step: Pre-CRRP evaluations

During this step, the following four evaluations/tests were performed on the same day in the morning, and in the following order: anthropometric data, spirometry test, 6MWT, and HGS. The 6MWT and the HGS were performed on patients not wearing facemask.

Anthropometric data e.g., age, height (cm), weight (kg), and body mass index (BMI, kg/m^2^) were determined. The obesity status [underweight (BMI <18.5 kg/m^2^), normal weight (BMI: 18.5–24.9 kg/m^2^), overweight (BMI: 25.0–29.9 kg/m^2^), and obesity (BMI ≥30.0 kg/m^2^)] was noted (Tsai and Wadden, 2013).

The spirometry test was performed by an experiment technician using a portable spirometer (SpirobankG MIR, delMaggiolino 12500155 Roma, Italy), according to international guidelines (Miller et al., 2005). The collected spirometric data [i.e., (FVC, L), (FEV_1_, L), maximal mid-expiratory flow (L/s), and FEV_1_/FVC ratio (absolute value)] were expressed as absolute values and as percentages of predicted local values (Ben Saad et al., 2013).

The 6MWT was performed outdoors in the morning by one physician (*HBS* in the authors’ list), according to the international guidelines (Singh et al., 2014). The 6MWT was performed along a flat, straight corridor with a hard surface that is seldom traveled by others (40 m long, marked every 1 m with cones to indicate turnaround points). During the 6MWT, some data were measured at rest (_Rest_) and at the end (_End_) of the walk [e.g., dyspnea (visual analogue scale (VAS)), heart-rate, oxyhemoglobin saturation (SpO_2_, %); SBP and DBP (mmHg)], and the 6MWD (m, % of predicted value), and the number of stops were noted. For some 6MWT data, delta exercise changes (ΔExercise = 6MWT_End_ value minus 6MWT_rest_ value) were calculated [e.g., ΔSpO_2,_ Δheart-rate, ΔDBP, ΔSBP, Δdyspnea (VAS)]. The test instructions given to the patients were those recommended by the international guidelines (Singh et al., 2014). Heart-rate was expressed as absolute value (bpm) and as percentage of the predicted maximal heart-rate [predicted maximal heart-rate (bpm) = 208—(0.7 x Age)] (Tanaka et al., 2001). Heart-rate and SpO_2_ were measured *via* a finger pulse oximeter (Nonin Medical, Minneapolis, MN). The heart-rate_End_ (bpm) was considered as heart-rate target for lower limb exercise-training (Fabre et al., 2017). The predicted 6MWD and the lower limit of normal (LLN) were calculated according to local norms (Ben Saad et al., 2009). The 6-min walk work (i.e., the product of 6MWD and weight (Chuang et al., 2001; Carter et al., 2003)) was calculated. The VAS is an open line segment with the two extremities representing the absence of shortness of breath and the maximum shortness of breath (Sergysels and Hayot, 1997). Dyspnea (VAS) is evaluated by the physician from 0 (no shortness of breath) to 10 (maximum shortness of breath) (Sergysels and Hayot, 1997).

The HGS test measures the maximum-voluntary upper-limb muscle strength using an adjustable handgrip dynamometer (TKK5401®, Takei Scientific Instruments Co., Ltd., Niigata, Japan). The latter is a valid and reliable measure having a range of 5–100 kg of force, with increments of 1 kg (Cadenas-Sanchez et al., 2016). A brief demonstration and verbal instructions for the test were given to patients, and if necessary, the dynamometer was adjusted to the size of the hand. The measurements were taken in a standing position with the shoulder adducted and in neutral rotation, and the arms parallel but not in contact with the body. Participants were asked to tighten the dynamometer as hard as possible while exhaling. The test was repeated three times on each hand. The highest value of the three trials of the dominant hand was retained (Haidar et al., 2004), and it was expressed as absolute (kg) and relative (i.e., divided by weight) values.

#### 2.5.3 Third step: Exercise-training

Exercise-training consists of 12 sessions (i.e., three sessions/week for 4 weeks).

**FIGURE 3:**
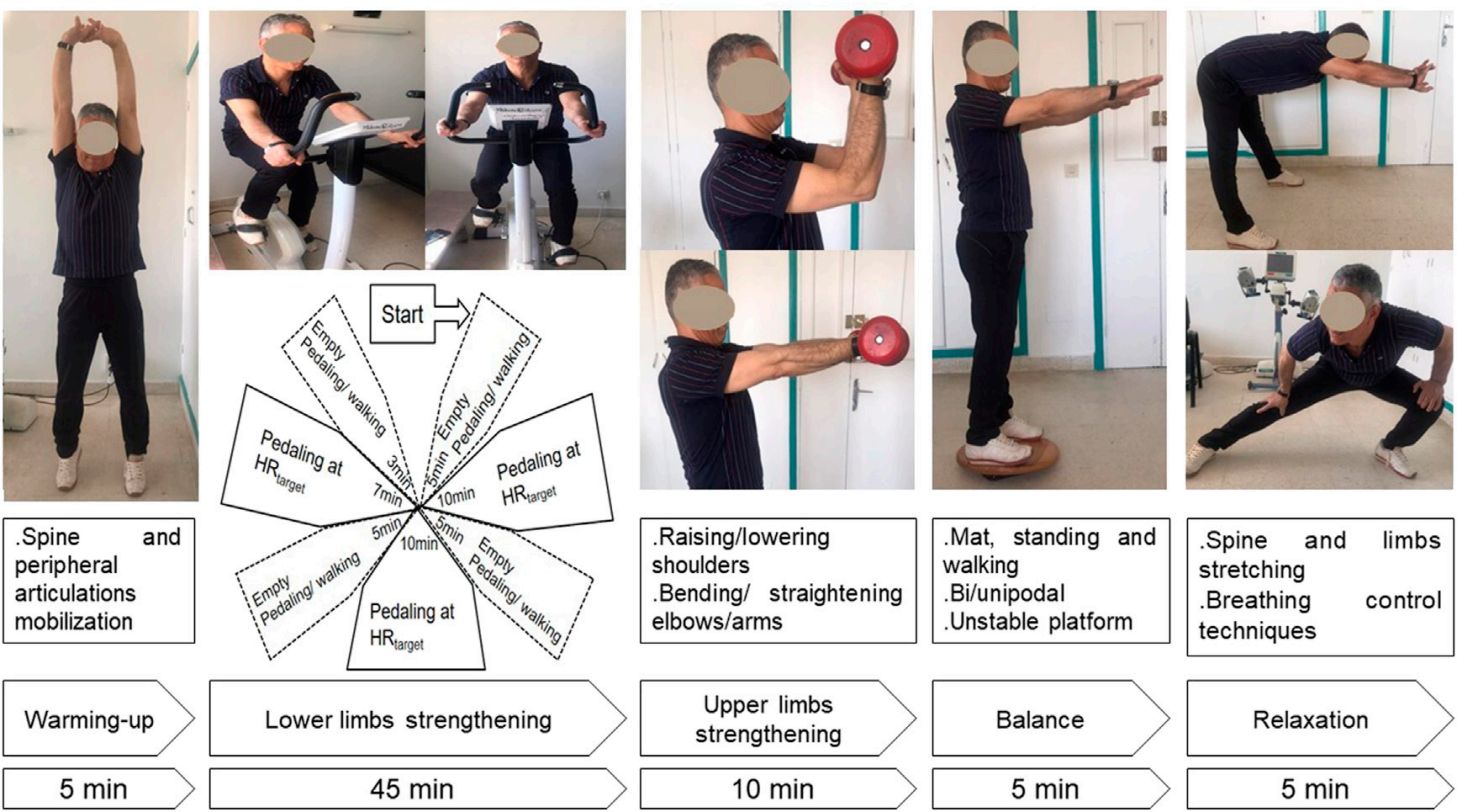
Description of an exercise training session. HR, heart-rate.

The duration of each session was 70 min. Exercise-training was performed in four groups of four or five patients. The typical exercise-training session included the following five items (Figure 3): warming-up for 5 minutes, lower limbs strengthening for 45 min, upper limbs strengthening for 10 min, balance posture and proprioception exercises for 5 minutes, and relaxation session for 5 minutes. During the *first item* (i.e., warming-up), light exercises were performed (i.e., walking slowly; mobilization of cervical, lumbar spine, and peripheral joints). During the *second item* (i.e., lower limbs strengthening), aerobic training on ergocycle was performed. The cycling intensity was standardized and personalized using a heart-rate monitor. As done in one previous similar study (Hermann et al., 2020), the heart-rate target was the heart-rate_End_ ± 5 bpm determined during the 6MWT. In patients with chronic respiratory conditions, the heart-rate at the first ventilatory threshold measured during a cardiopulmonary exercise test was comparable and correlated to the heart-rate determined at the end of the 6MWT (Fabre et al., 2017). The latter heart-rate target (i.e., heart-rate_End_) allows individualizing the training intensity for each patient, and therefore optimizing the physical and physiological benefits of the CRRP (Fabre et al., 2017). The heart-rate monitor alarms were set around the heart-rate target. The patients were asked to gradually reach their heart-rate targets during the first 5 minutes and to maintain pedaling for 10 min at this intensity. Then, they were asked to return to empty pedaling or walking at their own pace for 5 minutes. They were again asked to complete one cycle of 10 min of target heart-rate training and 5 minutes of active recovery (e.g., empty pedaling or walking at their own pace), then to complete the last cycle of 7 minutes of target heart-rate training and 3 minutes of active recovery. During the *third item* (i.e., upper limbs strengthening), various muscle groups of the upper limbs were performed in sets of ten repetitions (e.g., raising and lowering shoulders, shoulder blade stabilization, bending and straightening elbows, raising arms). These exercises were performed without load during the first exercise-training sessions, then with dumbbells of increasing weights along exercise-training (Ben Saad and Ben Abdelkrim, 2005). During the *fourth item*, several exercises were performed to improve balance posture, proprioception, coordination, and stability. Positions exercises (i.e., floor exercises, seated, and standing exercises) were varied between sessions. Exercises of increasing difficulty on a mat, static and dynamic standing and walking, bipodal, and then unipodal exercises on an unstable platform were performed along the exercise-training (Ben Saad and Ben Abdelkrim, 2005). During the *fifth item* (i.e., relaxation), several exercises involving spine and limbs stretching (e.g., standing stretch, cat back exercises, sphinx position) and breathing exercises (e.g., controlled diaphragmatic breathing, coordination between inspiratory and expiratory times) were performed (Ben Saad and Ben Abdelkrim, 2005). During each exercise-training session, therapeutic education was carried out to strengthen the patients’ adherence to the lifestyle counseling provided during the pre-CRRP meeting (e.g., management of comorbidities and encouraging smoking cessation when applicable, psychological support, and nutritional counseling) (Ghram et al., 2022). All exercise-training items were performed on patients not wearing the facemask.

#### 2.5.4 Fourth step: Post-CRRP evaluation

During this step, similar evaluations/tests to the second step were performed.

#### 2.5.5 Fifth step: Post-CRRP meeting

During this step, the following issues were tackled: patients’ feedback, questionnaire (as conducted during the first step, except the general questionnaire), checking the results of post-CRRP evaluations, and advising patients to continue exercise-training.

### 2.6 Applied definitions for the submaximal exercise data

1) Abnormal 6MWD: 6MWD < LLN (Singh et al., 2014);
2) Clinically significant desaturation: ΔSpO_2_ > 5 point (Ben Saad et al., 2009; Ben Saad et al., 2014; Ben Saad et al., 2015; Ben Saad, 2020);
3) Walk intolerance signs: clinically significant dyspnea [i.e., dyspnea_End_ (VAS) > 5/10] (Sergysels and Hayot, 1997; Ben Saad et al., 2014), and/or stopping during the 6MWT (Ben Saad et al., 2009; Ben Saad et al., 2014; Ben Saad et al., 2015; Ben Saad, 2020).

### 2.7 Statistical analysis

Quantitative and categorical data were presented as means ± standard deviation (95% confidence interval) and number (%), respectively. For each quantitative data (i.e., dyspnea (mMRC and VAS), weight, BMI, HGS, 6MWD, 6-min walk work, heart-rate, SpO_2_, SBP, DBP, and ΔExercise), a ΔCRRP was calculated. The Wilcoxon matched pairs test and the one-sided chi-2 test were used to compare the quantitative and categorical data pre- and post- CRRP, respectively. CRRP was considered “efficient” if the means of ΔCRRP for 6MWD and dyspnea (mMRC) exceeded the recommended MCIDs [i.e., 30 m for 6MWD (Singh et al., 2014)) and one point for dyspnea (Crisafulli and Clini, 2010)]. All statistical procedures were performed using statistical software (StatSoft, Inc. (2011). STATISTICA, version 12). The significance level was set at *p* < 0.05.

## 3 Results

An initial sample of 68 patients was recruited. After the application of the inclusion/non-inclusion criteria, 18 patients were retained. Four patients withdrew during CRRP. Fourteen patients (age: 61 ± 4 years) completed the full CRRP and tests evaluations (Figure 1).

Table 1 details the patients’ characteristics. The profile of COVID19 patients was characterized by high frequencies of overweight and obesity (*n* = 13/14; 93%), level-2 chest computed-tomography at admission (*n* = 10/14; 71%), and smoking (*n* = 9/14; 64%). The two most frequent medical comorbidities were diabetes-mellitus and arterial-hypertension.

**TABLE 1:** Initial descriptive data of patients with coronavirus disease 2019 (COVID19) (*n* = 14).

Data	Unit	Value
Anthropometric and medical data		
Age	Year	61 ± 4 (59–64)
Height	cm	170 ± 4 (167–172)
Weight	kg	89 ± 16 (80–99)
Body mass index	kg/m^2^	31.0 ± 5.2 (28.0–34.0)
Obesity status	Normal	1 (7)
	Overweight	7 (50)
	Obesity	6 (43)
Smoking status and data	Yes	9 (64)
	Pack-year	24 ± 18 (8–39)
Medical history	Diabetes-mellitus	8 (57)
	Arterial-hypertension	6 (43)
	Chronic obstructive pulmonary disease	5 (36)
	Dyslipidemia	3 (21)
	Dysthyroidism	1 (7)
	Coronary heart-disease	0 (0)
COVID19 data and severity classification		
Period before the cardiorespiratory rehabilitation program	Days	83 ± 30 (65–100)
Hospital stay	Days	17 ± 7 (10–17)
Chest computed-tomography severity classification	Extensive	4 (29)
	Severe	10 (71)
Clinical severity classification	Moderate	3 (21)
	Severe	11 (79)

Quantitative and categorical data were mean ± standard deviation (95% confidence interval) and number (%), respectively.

Table 2 illustrates the impact of CRRP on dyspnea, anthropometric, spirometric, and HGS data. Dyspnea (mMRC) was improved by 1.79 points, which exceeds the MCID of one point. FEV_1_ was improved by 110 ml (3.29%).

**TABLE 2:** Impact of CRRP on dyspnea, and anthropometric, spirometric and HGS data of patients with coronavirus disease 2019 (*n* = 14).

Data	Unit/Category	Pre-CRRP	Post-CRRP	ΔCRRP	*p*-value
Dyspnea	mMRC, point	2.07 ± 0.73 (1.65–2.49)	0.29 ± 0.47 (0.02–0.56)	−1.79 ± 0.80 (−2.25 to −1.32)	0.0009*
Weight	kg	89 ± 16 (80–99)	89 ± 16 (79–98)	−0.79 ± 2.19 (−2.05 to 0.48)	0.2845
BMI	kg/m^2^	31.0 ± 5.2 (28.0–34.0)	30.7 ± 5.2 (27.7–33.7)	−0.27 ± 0.78 (−0.72 to 0.18)	0.2845
Obesity status	Normal	1 (7)	0 (0)	—	0.3086
	Overweight	7 (50)	8 (57)	—	0.7049
	Obesity	6 (43)	6 (43)	—	—
FEV_1_	l	2.89 ± 0.64 (2.52–3.26)	3.01 ± 0.71 (2.60–3.42)	0.11 ± 0.18 (0.01–0.22)	0.0354*
	%	81 ± 17 (71–90)	84 ± 19 (73–95)	3.29 ± 4.97 (0.42–6.15)	0.0280*
FVC	l	3.68 ± 0.55 (3.36–3.99)	3.85 ± 0.74 (3.42–4.28)	0.17 ± 0.33 (−0.02–0.37)	0.0652
	%	88 ± 12 (81–94)	92 ± 16 (82–101)	4.00 ± 7.96 (-0.60–8.60)	0.0652
FEV_1_/FVC	Absolute value	0.78 ± 0.10 (0.72–0.83)	0.77 ± 0.10 (0.71–0.83)	−0.01 ± 0.03 (−0.02 to 0.01)	0.2787
MMEF	l/s	3.11 ± 1.33 (2.35–3.88)	3.17 ± 1.42 (2.35–3.98)	0.05 ± 0.34 (−0.15–0.25)	0.6832
	%	65 ± 27 (49–80)	66 ± 29 (49–82)	1.00 ± 7.21 (−3.16–5.16)	0.6378
HGS	Absolute value (kg)	36 ± 6 (33–40)	39 ± 6 (35–42)	2.35 ± 8.01 (−2.028–6.98)	0.6377
	Relative value	0.41 ± 0.08 (0.37–0.46)	0.45 ± 0.10 (0.39–0.50)	0.03 ± 0.10 (−0.02–0.09)	0.7298

BMI, body mass index; CRRP, cardiorespiratory rehabilitation program; FEV_1_, forced expiratory volume in one second; FVC, forced vital capacity; HGS, handgrip-strength; MMEF, maximal mid expiratory flow; mMRC, modified medical research council; %, percentage of predicted value. ΔCRRP, post-CRRP, value minus pre-CRRP, value. Quantitative and categorical data were mean ± standard deviation (95% confidence interval) and number (%), respectively. *p-value <0.05 (Wilcoxon matched pairs test or one-sided chi-2, test): pre-CRRP, vs. post-CRRP.

Table 3 illustrates the impact of CRRP on submaximal exercise data. The 6MWD increased by 35 m, which is higher than the MCID of 30 m. Nine patients (64.3%) increased their 6MWD by more than 35 m, and the number (%) of COVID19 patients with abnormal 6MWD decreased from 3 (21%) to 0 (0%). The 6-min walk work increased by 2,448 mkg. The heart-rate_Rest_ (bpm, %) decreased by seven bpm (5%), and DBP_Rest_ decreased by 6 mmHg.

**TABLE 3:** Impact of CRRP on submaximal exercise data of patients with coronavirus disease 2019 (*n* = 14).

Data	Unit/Category	Pre-CRRP	Post-CRRP	ΔCRRP	*p*-value
6-min walk distance	m	571 ± 53 (540–602)	606 ± 44 (581–631)	35 ± 42 (11–60)	0.0131*
	%	95 ± 9 (90–100)	102 ± 7 (98–106)	7 ± 8 (3–11)	0.0088*
	< LLN	3 (21)	0 (0)	—	0.0350*
6-min walk work	mkg	50,974 ± 10,052 (45,170–56,778)	53,442 ± 8,406 (48,569–58,276)	2,448 ± 3,925 (182–4,715)	0.0480*
HR (bpm)	HR_Rest_	78 ± 10 (72–84)	71 ± 12 (64–78)	−7 ± 9 (−13 to −2)	0.0175*
	HR_End_	118 ± 27 (103–134)	118 ± 27 (102–133)	−1 ± 32 (−19 to 18)	0.9250
	ΔExercise	40 ± 26 (25–55)	47 ± 22 (34–60)	6 ± 30 (−11–24)	0.4512
HR (%)	HR_Rest_	46 ± 6 (42–49)	41 ± 6 (37–45)	−5 ± 5 (−8 to −2)	0.0068*
	HR_End_	69 ± 16 (60–78)	69 ± 15 (60–78)	−1 ± 19 (−12 to 10)	0.8506
	ΔExercise	24 ± 15 (15–32)	28 ± 13 (20–35)	4 ± 18 (-6–14)	0.4326
SpO_2_ (%)	SpO_2Rest_	96 ± 2 (95–97)	96 ± 2 (94–97)	−0 ± 3 (−2 to 1)	0.3882
	SpO_2End_	94 ± 5 (91–97)	94 ± 8 (89–99)	-0 ± 4 (-3 to 2)	0.7897
	ΔExercise	−2 ± 5 (−5 to 1)	−2 ± 7 (−6 to 3)	0 ± 5 (−3 to 3)	0.6566
	Desaturation	2 (14)	1 (7)	—	0.2729
Dyspnea (VAS)	Dyspnea_Rest_	1 ± 2 (0–2)	1 ± 1 (0–2)	−0 ± 2 (−1 to 1)	0.7353
	Dyspnea_End_	3 ± 1 (2–3)	3 ± 2 (1–4)	0 ± 2 (-1 to 1)	0.9291
	ΔExercise	1 ± 1 (1–2)	2 ± 1 (1–3)	0 ± 2 (−1 to 1)	0.7221
	Dyspnea_End_ > 5	0 (0)	1 (7)	−	0.1568
SBP (mmHg)	SBP_Rest_	139 ± 14 (131–147)	134 ± 11 (127–140)	−6 ± 14 (−14 to 2)	0.1535
	SBP_End_	155 ± 16 (146–165)	151 ± 13 (144–159)	−4 ± 14 (−12 to 4)	0.3590
	ΔExercise	16 ± 11 (9–23)	18 ± 11 (11–24)	2 ± 12 (−5–9)	0.6784
DBP (mmHg)	DBP_Rest_	85 ± 8 (80–89)	79 ± 9 (73–84)	−6 ± 10 (−12 to −0)	0.0454*
	DBP_End_	84 ± 12 (77–90)	84 ± 11 (77–90)	0 ± 11 (−6 to 6)	0.9165
	ΔExercise	−1 ± 9 (−6 to 4)	5 ± 13 (-3–13)	6 ± 17 (−4–16)	0.2635

CRRP, cardiorespiratory rehabilitation program; DBP, diastolic blood pressure; _End_, at the end of the 6-min walk test (6MWT); HR, heart-rate; LLN, lower limit of normal; _Rest_, at rest before the 6MWT; SBP, systolic blood pressure; SpO_2_, oxyhemoglobin saturation; VAS, visual analog scale; 6MWD, 6-min walk distance; %, percentage of predicted value. ΔCRRP, post-CRRP, value minus pre-CRRP, value. ΔExercise = _End_ of exercise value minus _Rest_ value. Quantitative and categorical data were mean ± standard deviation (95% confidence interval) and number (%), respectively. *p-value<0.05 (Wilcoxon matched pairs test or one-sided chi-2, test): pre-CRRP, vs. post-CRRP.

## 4 Discussion

The present Tunisian study demonstrated that the CRRP improves the submaximal exercise capacity of post-COVID19 patients. For instance, the 6MWD improved by 35 m, which exceeds the MCID of 30 m, and the dyspnea (mMRC) improved by 1.78 point, which exceeds the MCID of one point. To the best of the authors’ knowledge, this is the first North-African study investigating the impact of CRRP on post-COVID19 patients. The methodology and main outcomes of some similar studies including a single group of COVID19 patients (Hermann et al., 2020; Betschart et al., 2021; Bouteleux et al., 2021; Daynes et al., 2021; Gloeckl et al., 2021; Piquet et al., 2021; Puchner et al., 2021), and case-control studies (Liu K. et al., 2020; Spielmanns et al., 2021) are detailed in Tables 4, 5, respectively.

**TABLE 4:** Methodology and main outcomes of some studies including a single group of COVID19 patients, and aiming at evaluating the impacts of CRRP on COVID19 patients.

1st author (Yr) [country]	a. Study design (type CRRP) b. Participants, N (male) c. Age (Yr)	Comorbidities (%)	Characteristics of CRRP program		Main outcomes	Summary of findings
			Components	a. Frequency b. Duration c. Period between the COVID19 diagnosis and CRRP starting (D) d. Other details		
Hermann et al. (2020), [Switzerland]	a. Interventional study (rehabilitation unit). b. 28 (14), Ventilated: 12, Not ventilated:16. c. Ventilated: 64 ± 9^a^, Not ventilated: 67 ± 10^a^	Ventilated: AH: 41.7, DM: 33.3, CKD: 25, dyslipidemia: 16.7, CHD: 8.3. Not ventilated: AH: 56.3, COPD: 37.5, dyslipidemia: 25, CHD: 18.8, DM: 18.8, CKD: 12.5, stroke: 6.3	**ET**. ACE (walking/cycling). Strength training. **Education**. Coping skills. Nutrition interventions. Activities of daily living	a. 5–6 D/W b. 25–30 sessions c. NR d. 2 D after being asymptomatic and 10 D after onset of infection	Spirometry, 6MWT	Improve in 6MWD*
Betschart et al. (2021), [Switzerland]	a. Pilot study (outpatient) b. 12 (8) c. 61 (26-84)^b^	CHD: 50, CKD: 42, AH: 25, malignancy: 25, CLuD: 16, internal disease: 16, DM: 8, obesity: 8, polyneuropathia: 8	**ET**. ACE training: 30 min RT: 30–40 min. **Education and physical activity coaching**	a. 2 D/W b. Minimum number of sessions = 16 c. 41.5 (21–73)^b^	6MWT	Improve in 6MWD*
Gloeckl et al. (2021), [Germany]	a. Prospective observational cohort study (rehabilitation unit) b. 50 (22), Mild/moderate: 24 (4), Severe/critical: 26 (18) c. Mild/moderate: 52 (47-56)^c^, Severe/critical: 66 (60-71)^c^	Mild/moderate: OSA:38, CLuD:30, AH:21, obesity:21, dyslipidaemia:13, CHD:5, DM:5 Severe/critical: AH:62, dyslipidaemia:38, OSA: 35, CHD:27, DM:23, CRD:23, obesity:19, CLuD:19, stroke:4	**ET**. ACE: 10–20 min. Strength training: 30 min. **Education** Respiratory physiotherapy. Activities of daily living training. Relaxation techniques. Occupational therapy. Psychological support. Nutritional counselling	a. 5 D/W. b. 3 W. c. Mild/moderate: 178 (127-217)^c^, Severe/critical: 61 (40–108)	mMRC, spirometry, DLCO, HGS, 6MWT, ESWT, 5rep STST	Mild/moderate: improve in FVC^α^, FEV_1_^α^, 6MWD^α^. Severe/critical: improve in HGS^β^, mMRC^β^, FVC^β¥^, FEV_1_^β¥^. 6MWD^β¥^, ESWT^β^. 5rep STST^β^
Daynes et al. (2021), [United Kingdom]	a. Observational study (outpatient). b. 30 (16). c. 58 ± 16^a^	Asthma: 10, COPD: 3	**ET**. Aerobic exercise (walking/treadmill based). Strength training. **Education**. .educational discussions with handouts	a. 2 D/W. b. 6 W. c. 125 ± 54^a^	CAT, ISWT, ESWT	Improve in CAT*. Improve in ISWT* and ESWT*
Puchner et al. (2021), [Austria]	a. Observational multicenter study (rehabilitation unit). b. 23 (16). c. 57 ± 10^a^	CHD:48, endocrine disease: 48, AH:39, DM:26, CLuD:22, CKD: 13, asthma: 13, malignancy: 13, immunodeficiency:13, CLiD: 9, hypercholesterolemia: 9, COPD: 4	**ET**. (25–50 min each session). Respiratory muscle training. Endurance and strength training. Passive therapy session (e.g., massages). Mobilization and breathing perception therapy. **Education**. Speech therapy and swallow evaluation. Occupational herapy . Neuropsychological therapy. Nutritional counseling	a. At least 3 W. b. 24 ± 5^a^D. c. 44 (13)^c^	Plethysmography, DLCO, MIP, 6MWT	Improve in FVC*, FEV_1_*, TLC*, DLCO*, MIP*. Improve in 6MWD*
Bouteleux et al. (2021), [France]	a. Observational longitudinal study (outpatient). b. 39 (17), PFS: 29 (11), NPFS: 10 (6). c. 48 ± 15^a^	No comorbidities	**ET**. (90 min each session). Aerobic exercise. Strength training. Specific controlled ventilation techniques	a. 3/W. b. 66 (26-110)^c^ D. c. 73 (34-178)^c^	mMRC, spirometry, hyperventitlation syndrome provocation test, Nijmegen score, 6MWT, 3min-STS	Improve in mMRC* and FVC*. Improve in 6MWD* and 3min-STS*
Piquet et al. (2021), [France]	a. Retrospective study (rehabilitation unit). b. 100 (66). c. 66 ± 22^c^	AH: 48, DM: 29, obesity: 17, CKD: 13, stroke: 9, immunodeficiency: 3, CHD: 1	**Respiratory rehabilitation**. Controlled diaphragmatic breathing. **ET**. ACE (bicycle ergometer). Motor strengthening (body weight exercises, elastics, weights). **Education**. Occupational therapy. Speech therapy. Psychological therapy. Nutritional counseling	a. 2sessions/D. b. 5 D/W, 10 ± 5^a^ D. c. 20 ± 10^c^	HGS, 10full-STS	Improve in HGS*. Improve in 10full-STS*

ACE, aerobic cycle endurance; AH, arterial-hypertension; BI, barthel index dyspnea; CAT, COPD, assessment test; CHD, coronary heart disease; CKD, chronic kidney disease; CLiD, chronic liver disease; CLuD, chronic lung disease; COPD, chronic obstructive pulmonary disease; COVID19, coronavirus disease 2019; CRRP, cardiorespiratory rehabilitation; D, day; DLCO, diffusing capacity of the lung for carbon monoxide; DM, diabetes-mellitus; ESWT, endurance shuttle walk test; ET, exercise-training; FEV_1_, forced expiratory volume in one second; FVC, forced vital capacity; HGS, handgrip strength; Min, minute; MIP, maximal inspiratory pressure; mMRC, modified medical research council dyspnea scale; N, number; NA, not-applied; NPFS, no prolonged functional sequelae; NR, not-reported; OSA, obstructive sleep apnea; PFS, prolonged functional sequelae; RT, resistance training; TLC, total lung capacity; VAS, visual analogue scale; W, week; Yr, year; 3min-STS, 3 min sit-to-stand test; 5rep-STST, five repetitions sit-to-stand test; 10full-STS, 10 full sit-to-stands test; 6MWT, 6-min walk test; 6MWD, 6-min walk distance; Data were: ^a^Mean ±SD; ^b^Median (minimum-maximum); ^c^Median (interquartile range). *p < 0.05: pre-CRRPvs. after CRRP. For the study of Gloeckl et al. (2021): ^α^p<0.05 pre-CRRP, vs. post-CRRP, for the same group mild/moderate; ^β^p<0.05 pre-CRRP, vs. post-CRRP, for the same group severe/critical. ^¥^ *p* < 0.05 between-group difference mild/moderate vs. severe/critical for the same period.

**TABLE 5:** Methodology and main outcomes of some case-control studies aiming at evaluating the impact of CRRP on COVID19 patients.

1st author (Yr) [country]	a. Study design (type CRRP). B. Participants, N (male. c. Age (Yr))	Comorbidities (%)	Characteristics of CRRP program		Main outcomes	Summary of findings: Comparison
			Components	a. Frequency. b. Duration. c. Period between the COVID19 diagnosis and CRRP starting (D)		
Liu K. et al. (2020), [China]	a. Randomized controlled trial (outpatient). b**.** 72 (49), Intervention: 36 (24), Control: 36 (25). **c.** Intervention: 69 ± 8^**a**^, Controls: 69 ± 8^**a**^	**Intervention:** AH: 28, DM: 25, osteoporosis: 22. **Controls:** DM: 25, AH: 22, osteoporosis: 17	**ET**. 0.10 min. Respiratory muscle training. Cough exercise. Diaphragmatic training. Stretching exercise. Home exercise	a. 2 D/W. b**.** 6 W. c. NR	.Spirometry, DLCO, 6MWT	**Intervention group:** Improve in FEV_1_^**α¥**^, FVC^**α¥**^, DLCO^**α¥**^, 6MWD^**α¥**^. **Controls:** No impact
Spielmanns et al. (2021), [Switzerland**]**	a. Interventional study (rehabilitation unit). b. 518 (263), PG:99 (57), LG:419 (206. c. PG: 68 ± 10^**a**^, LG: 69 ± 11^**a**^)	**PG:** AH:54, obesity:25, MSD:25, dyslipidemia:20, ND:20, CKD:19, CHD:18, malignancy:15, COPD:11. **LG:** NR	**ET**. ACE (cycling/treadmill): 10–30 min. Gymnastics: 45 min. Outdoor walking: 45 min.Strength training: 30 min. **Education**. Relaxation: 45 min . Respiratory therapy: 30 min	a. 5-6 D/W. b. 3 W, 25–30 sessions. c. 2 D after being asymptomatic and 10 D after onset of infection	Spirometry, 6MWT	**PG:** Improve in 6MWD^**Δ£**^. **LG:** Improve in 6MWD^**δ**^

ACE, aerobic cycle endurance; AH, arterial-hypertension; CHD, coronary heart disease; CKD, chronic kidney disease; COPD, chronic obstructive pulmonary disease; COVID19, coronavirus disease 2019; CRRP, cardiorespiratory rehabilitation; D, day; DLCO, diffusing capacity of the lung for carbon monoxide; DM, diabetes-mellitus; ET, exercise-training; FEV_1_, forced expiratory volume in one second; FVC, forced vital capacity; LG, lung diseases group; Min, minute; MSD, musculoskeletal disease; N, number; NA, not-applied; ND, neurological disease; NR, not-reported; PG, post-COVID19, group; W, week; Yr, year; 6MWD, 6-min walk distance; 6MWT, 6-min walk test; Data were ^a^Mean±SD. ^*^ *p* < 0.05. For the study of Liu K. et al. (2020): ^α^p<0.05 pre-CRRP, vs. post-CRRP, for the same group cases; ^¥^ *p* < 0.05 between-group difference cases vs. controls for the same period. For the study of Spielmanns et al. (2021): ^Δ^p<0.05 pre-CRRP, vs. post-CRRP, for the PG, group; ^δ^p<0.05 pre-CRRP, vs. post-CRRP, for the LG, group. ^£^ *p* < 0.05 between-group difference PG, vs. LG, for the same period.

### 4.1 Discussion of results

In this study, the increase in the main outcome (i.e., 6MWD) was both “statistically” and “clinically” significant (mean of 35 m, which exceeds the MCID of 30 m (Singh et al., 2014)). At the end of CRRP, no COVID19 patients had an abnormal 6MWD, and the heart-rate_Rest_ and DBP_Rest_ decreased by seven bpm (5%) and 6 mmHg, respectively (Table 3).

The mean increase in 6MWD reported in this study was intermediate with the values reported in the literature (Liu K. et al., 2020; Betschart et al., 2021; Gloeckl et al., 2021) (Tables 4, 5). The 35-m 6MWD mean was closer to these reported in some studies [e.g., 48 m for mild/moderate patients (Gloeckl et al., 2021), 50 m (Liu K. et al., 2020)], but it was lower than the values reported in some other studies [e.g., 88 m (Betschart et al., 2021), 124 m for severe/critical patients (Gloeckl et al., 2021), 130 m (Hermann et al., 2020), 176 m mean (Puchner et al., 2021)]. Similar to some studies (Hermann et al., 2020; Betschart et al., 2021; Gloeckl et al., 2021), the improvement in 6MWD noted in this study was “clinically significant.” Indeed, before CRRP, three patients had an abnormal 6MWD; and after CRRP, all patients had normal 6MWD (*p* = 0.03) (Table 3). This finding is inconsistent with the one reported in a German study (Gloeckl et al., 2021), where 79% of mild/moderate patients had an abnormal 6MWD after 3 weeks of inpatient rehabilitation. Because weight directly affects the work/energy required to perform the 6MWT (Holland et al., 2014; Singh et al., 2014), the 6-min walk work was calculated. The latter, which is the product of 6MWD and weight, provides a better estimate of the work required to perform the 6MWD than distance alone (Holland et al., 2014; Singh et al., 2014). In this study, since the 6-min walk work increased significantly (Table 3), and since there were no statistically significant changes in patients’ weight or BMI (Table 2), these confirm that the 6MWD improve is independent of changes in weight or BMI. To the best of the authors’ knowledge, no previous study investigated the change of the 6-min walk work before/after a CRRP in COVID19 patients. Additional studies are needed to better characterize the utility of 6-min walk work in rehabilitation programs of COVID19 patients.

The decrease in heart-rate_Rest_ and DBP_Rest_ noted in this study (Table 3) could have some clinical importance. It appears that increased heart-rate_Rest_ (after adjustment for fitness) is an independent risk factor for all-cause mortality in males (Aladin et al., 2014), and a previous report indicated that a 10-bpm increase in heart-rate_Rest_ may increase all-cause mortality by 17% (Aune et al., 2017). High blood pressure is among the most important modifiable risk factors for cardiovascular disease and death (Williams et al., 2018). After the CRRP, the mean DBP_Rest_ decreased from 85 to 79 mmHg, which is an interesting outcome since the 2018 European society of cardiology recommends an optimal DBP_Rest_ target between 70 and 80 mmHg for patients with all risk levels (Williams et al., 2018). The present decrease in heart-rate_Rest_ is comparable to previously reported findings in older adults indicating a beneficial effect for endurance-based exercise-training (Schmidt et al., 2014; Akwa et al., 2017) as well as combined exercise-training (Delecluse et al., 2004; Ammar et al., 2021) with a significant reduction of heart-rate_Rest_ ranging from 4.5 to eight bpm. Presumably, the present beneficial cardiac effects of twelve-CRRP sessions could be the result of an enhancement of the cardiovascular autonomic control, with possible modification in the sympathovagal balance (Gamelin et al., 2007; Ammar et al., 2021). However, the exact mechanisms require further investigation. Additionally, the reduced values of both heart-rate_Rest_ and DBP_Rest_ at post-CRRP could be explained by the improvement in fitness (Greenland et al., 1999), and/or sleep quality (Soler et al., 2013; Yuksel et al., 2014), and/or nutritional status (Singh et al., 2000). The aforementioned results have not been discussed in previous studies evaluating the effects of CRRP on 6MWT data in COVID19 patients (Table 4, 5).

In our study, while dyspnea (mMRC) and FEV_1_ were improved by 1.79 points and 110 ml, respectively, HGS remained unchanged (Table 2). The finding related to dyspnea is in line with previous reports (Table 4) indicating that CRRP improves perceived dyspnea [whatever its mode of evaluation; e.g., mMRC (Bouteleux et al., 2021; Gloeckl et al., 2021), chronic obstructive pulmonary disease assessment test (Daynes et al., 2021)], even in severe/critical COVID19 patients (Gloeckl et al., 2021). In our study, mMRC improvement was higher than the one point MCID (Crisafulli and Clini, 2010). To the best of the authors’ knowledge, this is the first study investigating MCID dyspnea after a CRRP in post-COVID19 patients. In these patients, improvement in perceived dyspnea is capital since dyspnea is significantly associated with higher mortality (Shi et al., 2020), and it is a predictive factor of reduced functional capacity (Wong et al., 2021).

Our results concerning spirometry data are comparable with those investigating the impact of CRRP on lung function data (Liu K. et al., 2020; Bouteleux et al., 2021; Gloeckl et al., 2021; Puchner et al., 2021) (Tables 4, 5). In the latter studies, at least one lung function parameter was improved [FEV_1_ (Liu K. et al., 2020; Gloeckl et al., 2021; Puchner et al., 2021), FVC (Liu K. et al., 2020; Bouteleux et al., 2021; Gloeckl et al., 2021; Puchner et al., 2021), total lung capacity (Puchner et al., 2021), diffusing lung capacity for carbon monoxide (DLCO) (Liu K. et al., 2020; Puchner et al., 2021)]. Concerning the improvement in FEV_1_, the 110-ml increase observed in our study was lower than the values reported in the literature [e.g., 200 ml (Puchner et al., 2021), 340 ml (Liu K. et al., 2020)]. The improvement in lung function data could be explained by the breathing exercises and the respiratory muscle training applied during CRRP (Liu K. et al., 2020; Gloeckl et al., 2021; Puchner et al., 2021). Improvement in lung function data, such as FVC and FEV_1_, is useful to improve risk stratification in patients with intermediate coronary heart disease (Lee et al., 2010). FVC is implicated in predicting cardiovascular events and thus mortality (Lee et al., 2010). In addition, even in healthy people, there is a positive correlation between spirometric data (e.g., FEV_1_ and FVC) and 6MWD (Ben Saad et al., 2009).

In our study, the HGS remained unchanged (Table 2). Our findings were inconsistent with those reported by two previous studies, where HGS improved by 3 kg (i.e., from 18 to 21 kg) (Piquet et al., 2021) or 5 kg (from 25 to 30 kg) (Gloeckl et al., 2021) (Table 4). The absence of improvement in HGS could be explained by the fact that the pre-CRRP HGS value (i.e., 37 kg) was in the norms [i.e., >27 kg (Cruz-Jentoft et al., 2019)], and by the inclusion of moderate to severe COVID19 patients (Table 1). In the two above-cited studies reporting improvement in HGS, patients were classified as severe or critical (Gloeckl et al., 2021), and both the pre- and the post- CRRP HGS values were below the norms (Piquet et al., 2021). HGS measurement is important since it is associated with frailty and with an increased risk of mortality (Cheval et al., 2021). Indeed, COVID19 survivors have an increased risk of acute sarcopenia (Welch et al., 2020) due to the loss of muscle mass, fiber denervation, neuromuscular junction damage, and upregulation of protein breakdown (Puthucheary et al., 2010).

### 4.2 Discussion of methods

The discrepancies noted between our results and these of some similar studies (Tables 4, 5) could be explained by at least seven points related to differences in:

1) Study designs: prospective observational cohort (Gloeckl et al., 2021) vs. case control (Liu K. et al., 2020) studies;
2) CRRP locations: outpatient (Betschart et al., 2021) vs. inpatient (Puchner et al., 2021) rehabilitation;
3) Some inclusion criteria such as inclusion of both males and females (Bouteleux et al., 2021; Daynes et al., 2021), which could have influenced the findings since COVID19 clinical data are sex-dependent (Marik et al., 2021); or inclusion of COVID19 patients having different ages (e.g., elderly (≥65 years) (Liu K. et al., 2020) vs. middle-aged (48 ± 15 years) (Bouteleux et al., 2021)) which could have influenced the findings (Liu Y. et al., 2020);
4) COVID19 patients’ profiles (e.g., no (Bouteleux et al., 2021), vs. several (Betschart et al., 2021; Gloeckl et al., 2021) comorbidities) and/or in the disease severity stages (e.g., mild/moderate vs. severe/critical) (Gloeckl et al., 2021);
5) CRRP’ components (e.g., exercise-training and education (Hermann et al., 2020; Betschart et al., 2021) vs. exercise-training alone (Bouteleux et al., 2021));
6) Durations and/or frequencies of CRRP (e.g., two sessions/week and 16 sessions (Betschart et al., 2021) vs. five sessions/week and three sessions (Gloeckl et al., 2021)); and
7) Time periods’ between the diagnosis of COVID19 and the start of CRRP [e.g., early rehabilitation for acute COVID19 (Puchner et al., 2021) vs. late rehabilitation for long-COVID19 (Daynes et al., 2021)].

### 4.3 Strengths and limitations

This study has three strong points. First, our study was conducted in an outpatient unit in a low-income country (e.g., Tunisia) and the different components were performed (i.e., exercise-training, education, and nutritional counseling). Second, our sample size was calculated according to a predictive equation (Serhier et al., 2020). Determination of the finest size is a central topic since it helps in avoiding an inadequate power to distinguish statistical effects (Mascha and Vetter, 2018), and it guarantees a representative sample to differentiate statistical significance (Serhier et al., 2020). Huge sample size is costly and exposes more participants to measures (Mascha and Vetter, 2018), but using insufficient participants may lead to lower “precision” in results. Third, both statistically and clinically significant approaches were applied. Nowadays, the statistically significant approach, with a “*p*-value” < 0.05 being considered significant, is disapproved (Yaddanapudi, 2016). The MCID of 30 m for the 6MWD (Singh et al., 2014) and one point for dyspnea (mMRC) were introduced (Crisafulli and Clini, 2010). For instance, in a German study (Gloeckl et al., 2021) (Table 4), it was demonstrated that post-CRRP dyspnea median (interquartile) value is significantly lower than the one measured pre-CRRP [2 (2-2) vs. 2 (1–2), *p* < 0.003]; but the zero mean difference between the two periods does not exceed the MCID of one point (Crisafulli and Clini, 2010).

The present study has two limitations. First, the lack of a control group is a major limitation. Indeed, the inclusion of a control group was reported only in few studies (Liu K. et al., 2020; Spielmanns et al., 2021) (Table 5). Several studies (Hermann et al., 2020; Betschart et al., 2021; Bouteleux et al., 2021; Daynes et al., 2021; Gloeckl et al., 2021; Piquet et al., 2021; Puchner et al., 2021) have included only one group (Table 4) and it was difficult to include a control group due to ethical considerations during the COVID19 pandemic. The lack of a control group did not allow us to “affirm” that our results are only attributable to CRRP. Indeed, one study reported that lung function data of most COVID19 patients improve spontaneously over 3-month period (Wu et al., 2021). Second, it would have been more interesting to explore the respiratory function using additional tests, such as plethysmography (Puchner et al., 2021), DLCO (Liu K. et al., 2020; Gloeckl et al., 2021; Puchner et al., 2021), and maximal inspiratory pressure (Puchner et al., 2021), and exercise data using a cardiopulmonary exercise test in order to determine the first ventilatory threshold. In COVID19 patients, the most frequent lung function impairment is altered DLCO (39%) (Torres-Castro et al., 2021). Due to the unavailability of equipment in our public health hospital, these examinations were not performed.

## 5 Conclusion

A 4-week CRRP in post-COVID19 patients improved dyspnea (mMRC), FEV_1_, 6MWD, 6-min walk work, resting heart-rate and DBP. CRRP has imposed itself as a standard of care for the treatment of post-COVID19 patients.

## Data Availability

The raw data supporting the conclusion of this article will be made available by the authors, without undue reservation.
